# Precision management of severe coronary artery calcification with concomitant left ventricular outflow tract obstruction: a case report

**DOI:** 10.3389/fmed.2026.1795850

**Published:** 2026-03-26

**Authors:** Wei Qi, Yazheng Zhang, Zhenzhen Wang, Kai Hou, Ting Li, Hongliang Cong

**Affiliations:** 1 Department of Cardiology, Tianjin Chest Hospital, Tianjin, China; 2 Department of Cardiology, Chest Hospital, Tianjin University, Tianjin, China; 3 Department of Respiratory and Critical Care Medicine, Tianjin Chest Hospital, Tianjin, China; 4 Department of Respiratory and Critical Care Medicine, Chest Hospital, Tianjin University, Tianjin, China; 5 Department of Ultrasound, Tianjin Chest Hospital, Tianjin, China; 6 Research Center, Pu’er People’s Hospital, Kunming University of Science and Technology Affiliated Hospital, Puer, China; 7 Department of Nuclear Medicine, Tianjin Chest Hospital, Tianjin, China

**Keywords:** CAC, HCM, IVUS, LVOTO, mavacamten, QFR

## Abstract

**Background:**

Severe coronary artery calcification (CAC) with hypertrophic cardiomyopathy (HCM) and left ventricular outflow tract obstruction (LVOTO) present complex diagnostic and therapeutic challenges.

**Case presentation:**

A 72-year-old man with exertional chest pain and dyspnea exhibited multivessel CAC (total score; 1,696), asymmetric septal hypertrophy, and dynamic LVOTO. Percutaneous coronary intervention (PCI) was performed on the right coronary artery (RCA) and left circumflex artery (LCX) guided by quantitative flow ratio (QFR) and intravascular ultrasound (IVUS). Left anterior descending artery (LAD) lesions showed preserved function but microvascular dysfunction; no intervention was performed. Cardiac magnetic resonance imaging confirmed HCM with segmental fibrosis and LVOTO.

**Intervention and outcome:**

Post-PCI, dual antiplatelet therapy was administered for 6 months, followed by aspirin monotherapy and oral mavacam (2.5 mg daily. After 4 months, the LVOT gradient decreased from 51 mmHg to 9–12 mmHg, with symptom resolution.

**Conclusion:**

Multimodal management combining coronary physiology, intravascular imaging, and targeted pharmacotherapy effectively relieved myocardial ischemia and LVOTO, thereby providing a reference for complex cases.

## 1 Introduction

Cardiovascular disease (CVD) is the leading cause of death worldwide, and coronary artery calcium (CAC), a marker of atherosclerosis, is strongly associated with the risk of clinical CVD (1, 2) and is a valuable tool for personalized cardiovascular care, guiding clinicians to tailor statin therapy based on individual CAC scores. Percutaneous coronary intervention (PCI) with angioplasty and stenting remains a critical and potentially lifesaving treatment option for coronary artery disease associated with severe calcification in cases involving proximal coronary thromboembolism (3, 4). Although uncommon, CAC with hypertrophic cardiomyopathy (HCM) is clinically significant and closely related to microvascular dysfunction in HCM, in which chronic myocardial ischemia promotes fibrosis and dystrophic calcification within the hypertrophied myocardium (5). Left ventricular outflow tract obstruction (LVOTO) is a key feature of obstructive hypertrophic cardiomyopathy (HOCM), defined as a peak instantaneous pressure gradient ≥30 mmHg across the left ventricular outflow tract (7). Symptoms include dyspnea, exertional chest discomfort, presyncope, or exertional syncope. LVOTO is an important risk marker in the European Society of Cardiology (ESC) HCM sudden cardiac death risk model. HCM presents with heterogeneous clinical manifestations and complex pathophysiology. Effective treatment strategies are available, including implantable defibrillators to prevent sudden death, medications, surgical myectomy to relieve outflow tract obstruction and heart failure symptoms, and pharmacological therapies to control atrial fibrillation and prevent embolic stroke (8). Mavacamten, a cardiac myosin inhibitor, promotes an energy-saving, superrelaxed state of myosin to reduce systolic and diastolic cross-bridge formation, sustainably lowering resting and valsalva-induced left ventricular outflow tract gradients, although real-world data on its effectiveness remain limited (9). Here, we present a case illustrating the comprehensive diagnostic and therapeutic management of a patient with a coronary artery high-resistance calcified lesion and LVOTO to provide a reference for precise diagnosis and treatment.

## 2 Case presentation

A 72-year-old man presented with a three-month history of intermittent exertional chest pain radiating to the left shoulder, arm, and posterior thorax accompanied by chest tightness and dyspnea. Coronary computed tomography and angiography revealed multivessel disease (total CAC score; 1,696), indicating severe coronary artery disease. For non-left main disease, a visually estimated diameter stenosis of ≥70%, and for left main disease a diameter stenosis of ≥50%, are commonly used to define significant stenosis and to guide revascularization strategies (10, 11). Significant stenosis was observed in the left anterior descending artery (LAD), left circumflex artery (LCX), and right coronary artery (RCA), with 95% of the RCA showing proximal-to-mid stenosis (Supplementary Figure S1). The LAD showed a maximal stenosis of about 70%, while the LCX had heavily calcified lesions with up to 90% stenosis. Overall, the coronary lesions were characterized by multi vessel involvement with severe calcification, tortuosity, and diffuse long-segment disease in a right-dominant coronary circulation. Given that the lesion in the proximal-to-mid RCA was the most critical and located near the ostium, and considering that the RCA supplied a larger myocardial territory compared with the LCX, the RCA lesion was considered the culprit lesion responsible for the patient’s angina. Therefore, revascularization of the RCA was prioritized to achieve the most significant improvement in myocardial ischemia. Two drug-eluting stents (3.0 × 33 mm and 3.5 × 25 mm) were successfully implanted into the RCA, achieving a postoperative quantitative flow ratio (QFR) of 0.91. The patient’s condition improved and was discharged.

**Figure 1:**
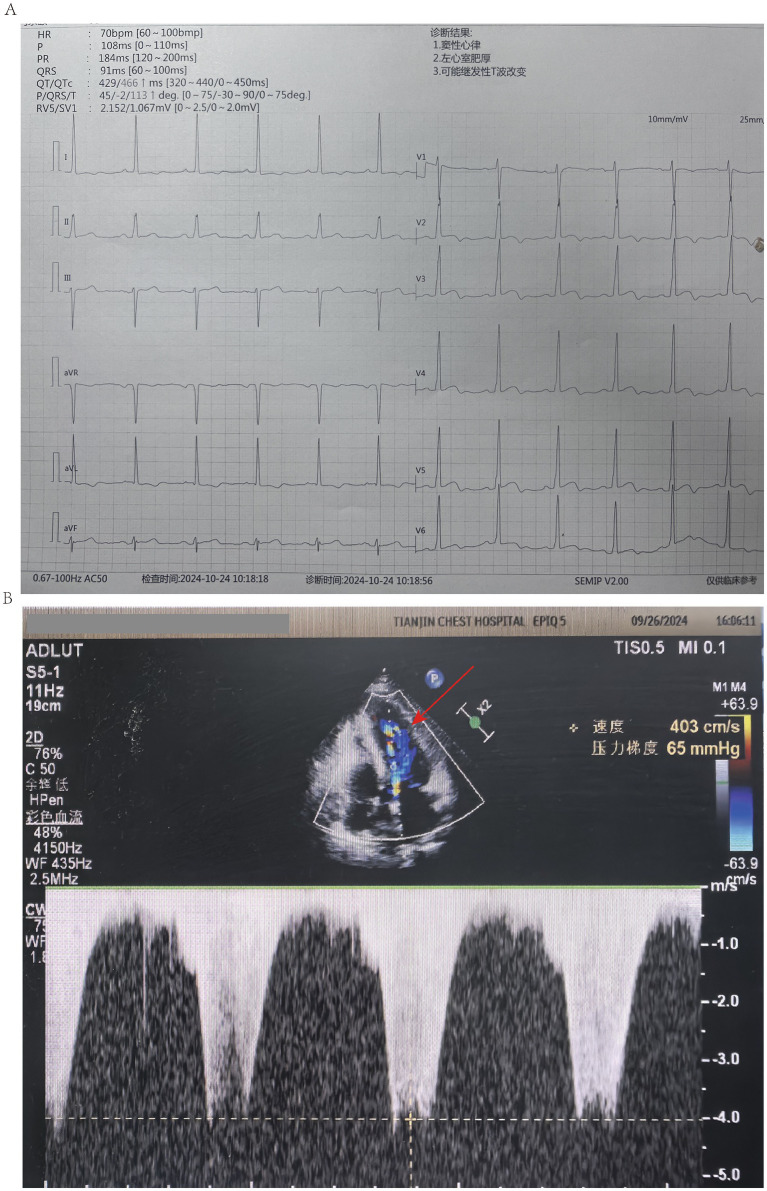
Electrocardiography and transthoracic echocardiography of the patient. **(A)** Electrocardiography. **(B)** Transthoracic echocardiography. Red arrow indicates left ventricular outflow tract (LVOT) obstruction.

One month post-discharge, the patient was readmitted because of recurrent exertional chest pain. Physical examination revealed sinus rhythm and blood pressure of 127/71 mmHg. Electrocardiography revealed ST-T wave changes suggestive of myocardial ischemia (Figure 1A). Transthoracic echocardiography revealed asymmetric hypertrophy of the mid-to-distal interventricular septum and apex (maximum thickness; approximately 23 mm), with speckled echogenicity, preserved systolic function, grade I diastolic dysfunction, and dynamic LVOTO (peak velocity; 403 cm/s, pressure gradient; 65 mmHg) (Figure 1B). Mild valvular regurgitation was observed, consistent with those of HOCM patients with Canadian Cardiovascular Society class III angina.

**Figure 2:**
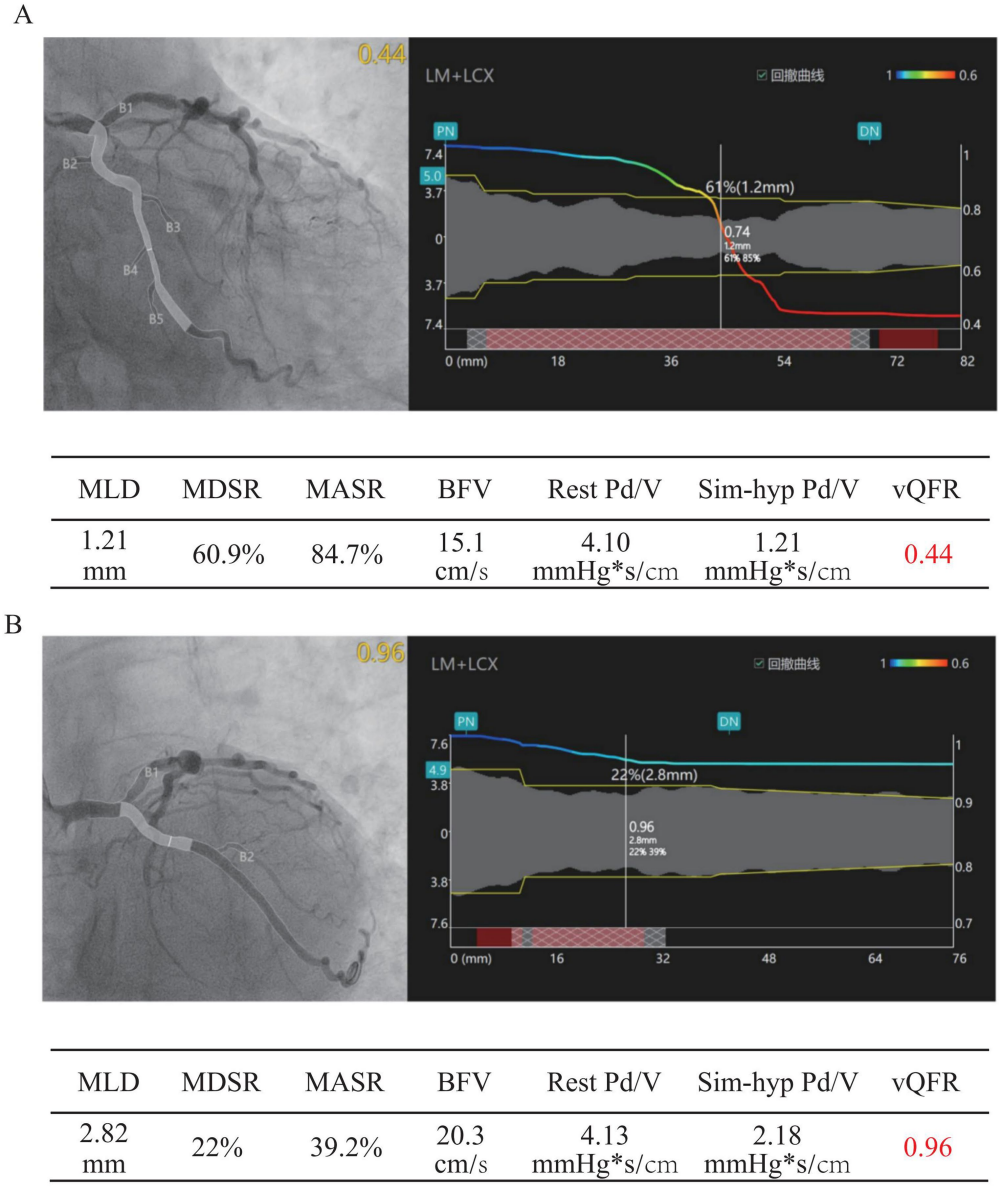
Coronary angiography results before and after stent implantation in the patient. **(A)** Coronary angiography before stent implantation showing multivessel disease with severe left circumflex artery (LCX) stenosis and heavy calcification. Quantitative flow ratio (QFR) measured 0.44, indicating hemodynamically significant stenosis requiring intervention. **(B)** Coronary angiography after stent implantation showing successful lesion modification. Two stents (2.5 × 28 mm and 3.5 × 24 mm) were implanted with post-dilation, achieving a QFR of 0.96. MLD, Minimum Lumen Diameter; MDSR, Minimal Diameter Stenosis Rate; MASR, Minimal Area Stenosis Rate; BFV, Blood Flow Velocity; Rest Pd/v, Resting Distal-to-Aortic Pressure Ratio (Pd/Pa); Sim-hyp Pd/v, Simulated Hyperemic Distal-to-Aortic Pressure Ratio (Pd/Pa); QFR, Quantitative Flow Ratio.

**Figure 3:**
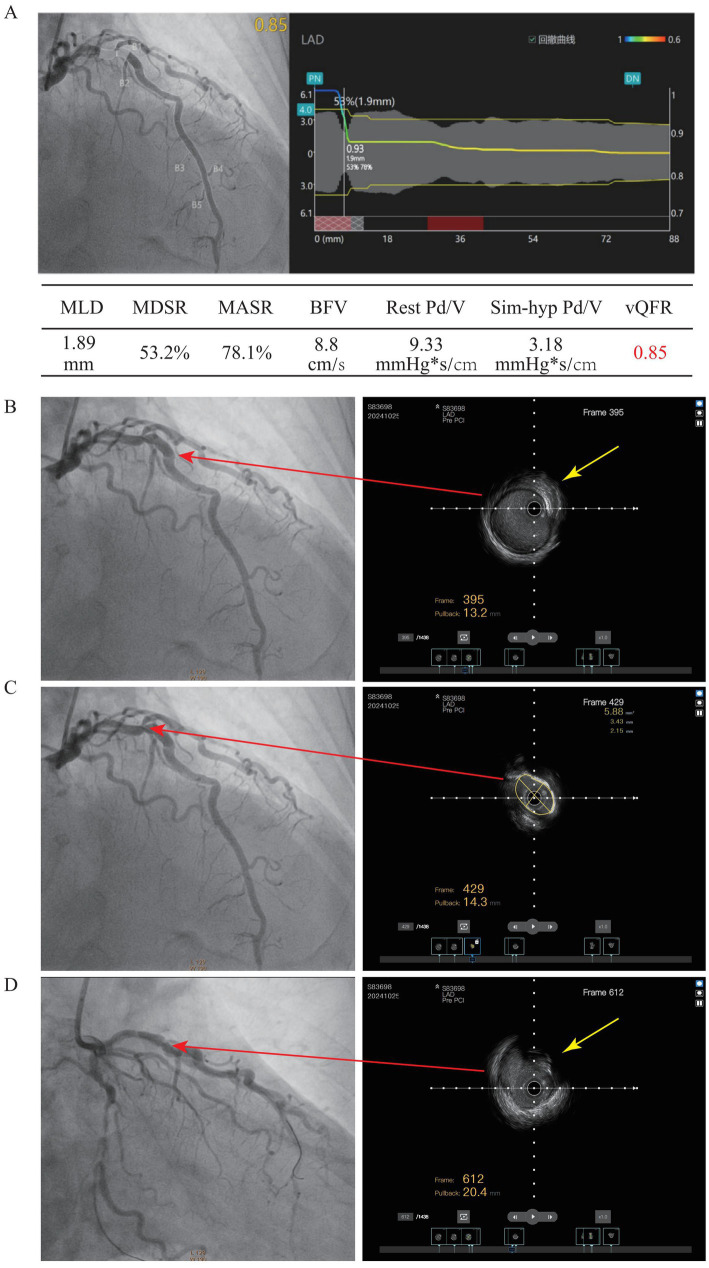
The coronary angiography and intravascular imaging with intravascular ultrasound (IVUS) images of left anterior descending artery (LAD). **(A)** The Coronary angiography and QFR of LAD; **(B)** Intravascular imaging with intravascular ultrasound (IVUS) at FRAME 395, **(C)** FRAME 429, **(D)** and FRAME 612. MLD, Minimum Lumen Diameter; MDSR, Minimal Diameter Stenosis Rate; MASR, Minimal Area Stenosis Rate; BFV, Blood Flow Velocity; Rest Pd/v, Resting Distal-to-Aortic Pressure Ratio (Pd/Pa); Sim-hyp Pd/v, Simulated Hyperemic Distal-to-Aortic Pressure Ratio (Pd/Pa); QFR, Quantitative Flow Ratio.

Coronary angiography revealed multivessel disease, with severe LCX stenosis and heavy calcification. QFR was 0.44, indicating hemodynamically significant disease (Figure 2A). Intravascular imaging with intravascular ultrasound (IVUS) revealed distal negative remodeling. After adequate predilation, the calcified lesion was modified. Two stents (2.5 × 28 mm and 3.5 × 24 mm) were implanted under IVUS guidance, with post-dilation (QFR; 0.96, Figure 2B). LAD angiography revealed multiple severe stenotic conditions, and interventional treatment was planned; however, after calculation, the QFR was 0.85 and index of microvascular resistance was 31.8 (Figure 3A), with preserved normal functional capacity, but coronary microvascular dysfunction (CMVD) was noted. IVUS revealed two severely stenotic segments with vessel folds (FRAME 395 and 612) (Figures 3B,D). The most severely stenotic segment demonstrated heavy calcification with a minimal lumen area (MLA) of 5.88 mm^2^, long diameter of 3.43 mm, and short diameter of 2.15 mm (FRAME 429) (Figure 3C), and PCI was deferred. Myocardial perfusion imaging demonstrated mild-to-moderate reversible ischemia in the RCA and LCX territories (approximately 20% of the left ventricle) and mild ischemia in the LAD territory (approximately 5%), consistent with the QFR and IVUS findings (Supplementary Figure S2).

**Figure 4:**
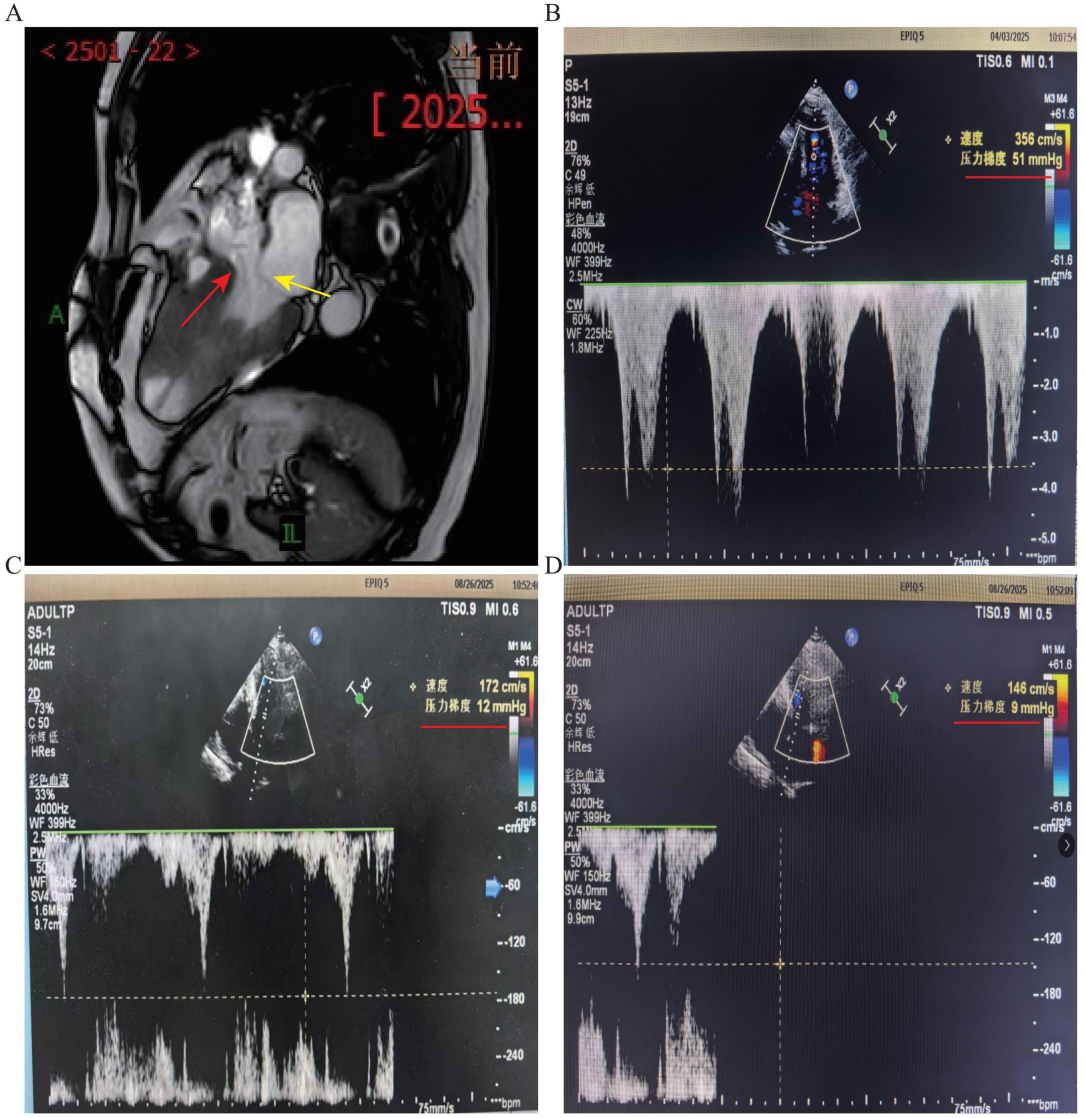
Cardiac magnetic resonance imaging and transthoracic echocardiography after PCI intervention in the RCA and LCX. **(A)** Post-intervention cardiac magnetic resonance imaging. Red and yellow arrows indicated the left ventricular hypertrophy and left ventricular outflow tract obstruction, respectively. **(B)** Transthoracic echocardiography after PCI in the RCA and LCX. **(C)** Resting transthoracic echocardiography after 4 months of mavacamten treatment. **(D)** Transthoracic echocardiography during the Valsalva maneuver.

Cardiac magnetic resonance imaging confirmed asymmetric left ventricular hypertrophy in the interventricular septum (29–30 mm), with apical thinning (4–5 mm), reduced systolic function, impaired diastolic compliance, thickened papillary muscles and trabeculae, and segmental myocardial fibrosis (Figure 4A and Supplementary Figure S3). Genetic testing excluded the possibility of Fabry disease. The final diagnosis was obstructive HCM with extensive segmental fibrosis, severe multivessel coronary calcification, LVOTO, and CMVD.

Post-PCI management included dual antiplatelet therapy (aspirin and clopidogrel) for 6 months, followed by aspirin monotherapy and mavacam 2.5 mg daily for HOCM. After 4 months, echocardiography showed complete resolution of LVOTO (resting peak velocity; 146 cm/s, pressure gradient; 9 mmHg, valsalva; 172 cm/s, 12 mmHg) (Figures 4B,C). The patient’s exertional chest pain and dyspnea resolved completely.

In patients with severe coronary calcification complicated by HOCM, a precision-based, multimodal management approach combining coronary physiology assessment, intravascular imaging, myocardial perfusion evaluation, and targeted pharmacotherapy can effectively relieve myocardial ischemia and LVOTO, achieving symptom resolution and functional improvement. This approach provides a reference for the precise diagnosis and management of complex cardiovascular cases.

## 3 Discussion

LVOTO restricts blood flow from the left ventricle and accounts for approximately 6% of all heart diseases. The resulting impairment of forward flow increases left ventricular afterload and may lead to left ventricular hypertrophy, dilatation, and heart failure (12). Hypertrophic cardiomyopathy and aortic stenosis can cause persistent hemodynamic obstruction of blood flow. Patients with hypertrophic cardiomyopathy undergoing trans catheter aortic valve replacement have poorer in-hospital outcomes, including cardiogenic shock, renal failure, and death (13). Previously, LVOTO was considered a hallmark of HCM, and cardiac catheterization-derived left ventricular pressure gradients were considered essential for diagnosis. However, as LVOTO is not present in all patients with HCM, non-invasive echocardiography with Doppler imaging has become the cornerstone of accurate HCM diagnosis and advanced the understanding of LVOTO mechanisms (14). In HCM, particularly apical HCM, myocardial hypertrophy and apical cavity obliteration lead to dynamic compression of intramyocardial small vessels, resulting in recurrent microvascular ischemia (15). This microvascular stress promotes myocardial fibrosis and endocardial scarring, which can ultimately progress to secondary endomyocardial fibrosis (EMF) with calcification. Thus, myocardial hypertrophy acts as the primary driver of microvascular dysfunction, while microvascular injury underlies the development of EMF and calcification, ultimately causing myocardial stiffness and impaired diastolic function, characteristic of this disease. Severe CAC significantly increases PCI complexity and risk, impeding device delivery and predisposing to complications such as vessel dissection, perforation, and stent underexpansion or malposition (16). IVUS studies have shown that severe CAC and the presence of calcified nodules are associated with higher rates of periprocedural myocardial infarction, major adverse cardiac events, and target lesion revascularization (6, 17, 18). These findings underscore the prognostic and procedural challenges of PCI in heavily calcified lesions. Interestingly, in a study of monozygotic twins with coronary artery calcification and hypertrophic cardiomyopathy, although both sisters had the same genetic susceptibility and similar lifestyles, they exhibited differences in multiple cardiac phenotypes including HCM, valvular calcification, and coronary artery dominance (19). In clinical practice, coronary computed tomography angiography (CCTA) is widely used as a noninvasive method for coronary artery evaluation (20, 21). However, in cases of severe coronary calcification, high-density calcium can produce beam-hardening and blooming artifacts, leading to blurred luminal boundaries and inaccurate estimation of stenosis severity, most commonly overestimation of luminal narrowing (22). Under these conditions, CCTA cannot reliably determine whether severe stenosis or occlusion is present beneath calcified plaques, nor can it accurately assess the functional significance of heavily calcified lesions. Therefore, when significant stenosis is accompanied by severe calcification, invasive coronary angiography combined with physiological assessment and intravascular imaging remains the gold standard for diagnosis and enables simultaneous interventional treatment when necessary (23, 24). Here, the patient had varying degrees of coronary artery stenosis in the LAD, RCA, and LCX. We prioritized revascularization of the culprit vessel, the RCA. Due to the complex, severely calcified and high-resistance nature of the RCA lesion, the procedure was technically challenging. Balloon pre-dilation, stent delivery, deployment, and post-dilation were all difficult, resulting in a relatively long procedural time and increased contrast use, which posed challenges to the patient’s procedural tolerance and safety. Considering the presence of multi vessel coronary artery disease and the potential risk of stent thrombosis despite adequate dual antiplatelet and anticoagulation therapy, we decided to terminate the procedure after successful RCA revascularization to ensure overall procedural safety. The patient subsequently received optimized medical therapy, including dual antiplatelet, lipid-lowering, and anti-anginal treatment with close clinical observation, along with oral Nicorandil (5 mg, three times daily) and Coenzyme Q10 (10 mg, three times daily). Although the patient’s symptoms improved after the RCA intervention, he continued to experience intermittent exertional chest pain radiating to the left shoulder, left arm, and posterior thorax, accompanied by chest tightness and dyspnea. Therefore, staged percutaneous coronary intervention of the LCX was planned to further relieve symptoms and to assess the physiological significance of the remaining coronary lesions. QFR combined with IVUS can effectively assess the severity of coronary stenosis and guide decisions on whether a patient should undergo PCI. A recent randomized trial showed that using QFR combined with IVUS to guide PCI effectively assessed stenosis severity, guided intervention, and significantly reduced the incidence of myocardial infarction, including lesions deemed non-significant by angiography that caused myocardial infarction during follow-up (25). In a comparative study on the diagnostic performance of moderate-to-severe calcified coronary lesions, improved QFR achieved a diagnostic sensitivity of 87.5% and specificity of 96.2% for coronary stenosis compared to the fractional flow reserve, effectively guiding subsequent patient management (26). IVUS is a valuable tool for guiding PCI as it assesses lesion morphology and helps reduce the risk of stent thrombosis. The hybrid Near-infrared spectroscopy (NIRS)-IVUS-QFR approach enables comprehensive evaluation by combining functional assessment with the anatomical and morphological characterization of plaques. Furthermore, integrating NIRS with IVUS enables tailored treatment strategies and optimizes lipid-lowering therapy in patients with thin-cap fibroatheroma lesions (27). Recent evidence supports physiology and imaging-guided PCI for improved outcomes (Supplementary Table S1). The FAVOR III China trial demonstrated that QFR-guided PCI reduced 2-year major adverse cardiovascular events (MACE) from 12.5% to 8.5% versus angiography-guided PCI. IVUS-guided PCI enhances stent expansion and lowers restenosis, target lesion failure, and MACE, while NIRS-IVUS hybrid imaging enables plaque characterization and optimized procedural planning beyond conventional angiography. Moreover, the RCA and LCX had QFR < 0.8, indicating the need for PCI, whereas the LAD had a QFR of 0.85. Subsequent assessment with IVUS confirmed that the degree of stenosis in the LAD was insufficient to cause myocardial ischemia. Therefore, no intervention was performed. According to current ESC guidelines, the revascularization strategy for patients with multivessel coronary artery disease should be individualized based on left ventricular ejection fraction, symptom burden, procedural risk, and multidisciplinary Heart Team evaluation, with either PCI or coronary artery bypass grafting considered as appropriate options (28). Meanwhile, the management of HCM, as recommended by both ACC/AHA and ESC guidelines, follows a stepwise approach based on symptom severity and the presence of LVOTO (29). In patients with persistent symptoms despite conventional medical therapy, treatment options may include septal reduction therapies or pharmacological strategies such as cardiac myosin inhibitors. Among these, myosin inhibitors, including mavacamten, have been recognized by current guidelines as an effective therapy for symptomatic obstructive HCM, with evidence demonstrating their ability to reduce left ventricular outflow tract gradients and improve clinical symptoms (29). Furthermore, in patients with LVOTO, mavacamten effectively reduced left ventricular outflow tract gradients >100 mmHg, achieving significant improvement within 1 week, while enhancing myocardial work parameters. After discontinuation of clopidogrel and administration of oral mavacamten (2.5 mg once daily), the patient’s pressure gradient decreased from 51 mmHg to 9–12 mmHg over 4 months, with complete resolution of chest tightness and shortness of breath (Figures 4B–D). This further confirmed the accuracy of our diagnosis and effectiveness of comprehensive treatment.

This study analyzed myocardial ischemia by integrating coronary angiography, QFR, IVUS, and emission computed tomography. PCI was performed on the RCA and LCX based on the QFR and IVUS findings, whereas three areas of vessel folding in the LAD were left untreated, demonstrating the superior performance of QFR over angiography in assessing calcified coronary stenosis. Targeted treatment with mavacamten was administered for HCM-related LVOTO, significantly improving myocardial ischemia symptoms. We hope that this case report provides valuable guidance for the management of patients with coronary calcification combined with HCM or LVOTO.

## Data Availability

The original contributions presented in the study are included in the article/Supplementary material, further inquiries can be directed to the corresponding author.

## Supplementary material

SUPPLEMENTARY FIGURE S1 Coronary angiography for the RCA.

SUPPLEMENTARY FIGURE S2 Myocardial perfusion imaging of the patient.

SUPPLEMENTARY FIGURE S3 Four-chamber cine images and Short-axis four-chamber LGE images. (A) The four chamber image shows asymmetric thickening of the left ventricular wall, with significant thickening of the interventricular septum. (B-D) Short-axis, four-chamber, and two-chamber delayed images show delayed enhancement of the sub endocardium in the mid-to-distal segments of the left ventricle and the apex.
