# Supplementary figures and images for: Precision management of severe coronary artery calcification with concomitant left ventricular outflow tract obstruction: a case report
Source: Front Med (Lausanne). 2026 Mar 26;13:1795850. doi: 10.3389/fmed.2026.1795850 (PMC13064543; doi:10.3389/fmed.2026.1795850)

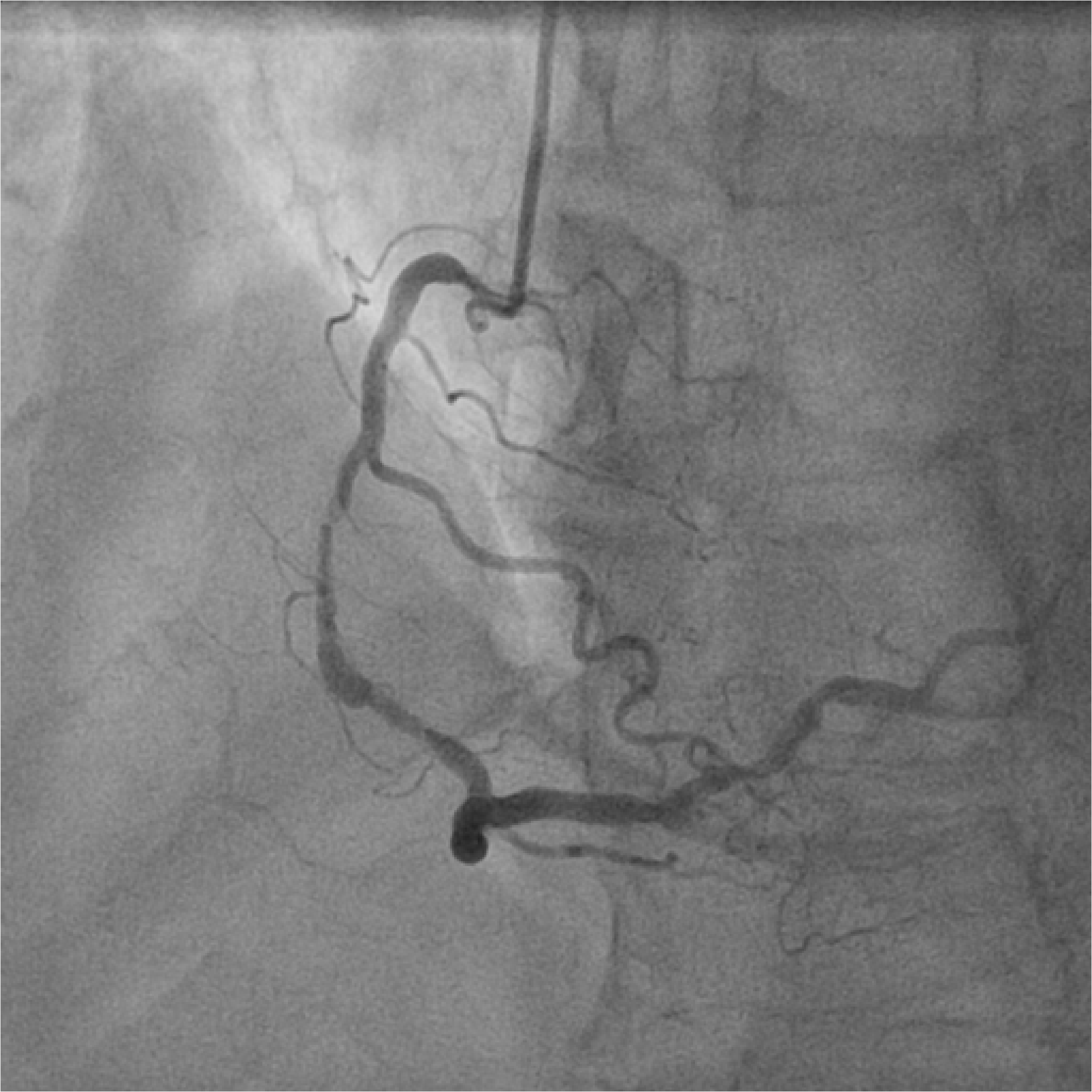

Supplement: SUPPLEMENTARY FIGURE S1 — Coronary angiography for the RCA. [file Image_1.TIF]

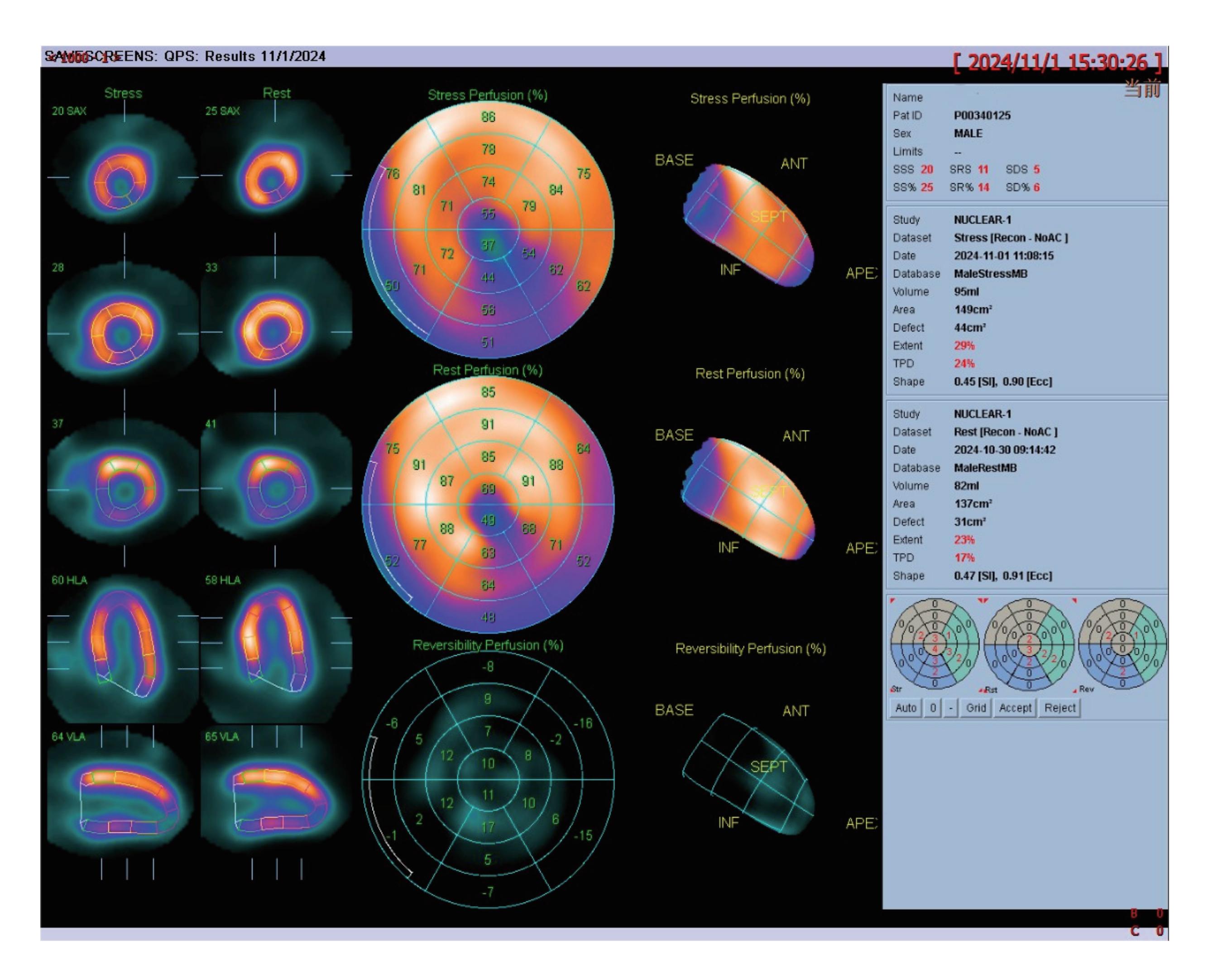

Supplement: SUPPLEMENTARY FIGURE S2 — Myocardial perfusion imaging of the patient. [file Image_2.JPEG]

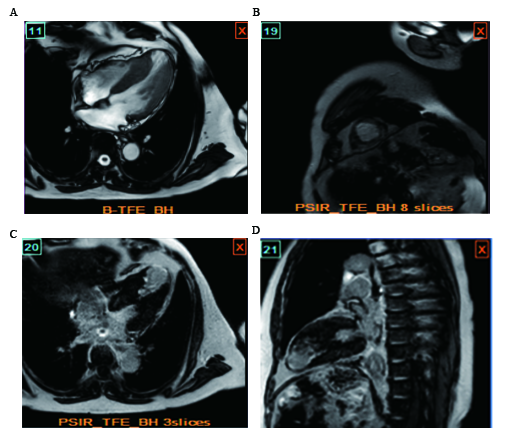

Supplement: SUPPLEMENTARY FIGURE S3 — Four-chamber cine images and Short-axis four-chamber LGE images. (A) The four chamber image shows asymmetric thickening of the left ventricular wall, with significant thickening of the interventricular septum. (B-D) Short-axis, four-chamber, and two-chamber delayed images show delayed enhancement of the sub endocardium in the mid-to-distal segments of the left ventricle and the apex. [file Image_3.TIF]
